# Extending the Age Range in Mammography Screening: A Benefit-Risk Assessment from a Radiation Protection Perspective

**DOI:** 10.1055/a-2674-5744

**Published:** 2025-08-28

**Authors:** Theresa Hunger, Elke Anna Nekolla, Eva Wanka-Pail, Katharina Stella Winter, Gunnar Brix

**Affiliations:** 1 39419 Medical and Occupational Radiation Protection, Federal Office for Radiation Protection, Neuherberg, Germany

**Keywords:** mammography, breast cancer, screening

## Abstract

**Background:**

Mammography screening programs (MSP) are established for women age 50 to 69 years in Germany and Europe. Some of the studies that build the evidence base for these programs also included women who were younger or older than this target population. The aim of our study was to assess whether screening also provides more benefit than harm to women outside the originally defined age range of the German MSP.

**Methods:**

A systematic review and meta-analysis of randomized controlled trials (RCT) was performed to assess overall and breast cancer mortality in women older than 70 years and women under 50 years. Radiation-associated age-specific lifetime attributable risks (LAR) were estimated based on a modified risk model of the BEIR Committee using current cancer and lifetime data for a female German population.

**Results:**

Two RCTs with 33,268 women age 70 years or older, and eight RCTs with 394,080 women age 39–49 years were included. The relative reduction in breast cancer mortality was 28% (risk ratio (RR) = 0.72; 95% confidence interval (CI): 0.54–0.95) and 18% (RR = 0.82; 95%-CI: 0.71–0.96), respectively. The proportion of overdiagnoses in older women is estimated at 19% and is higher than in younger women. Assuming biennial screening from below 50 to 69 years of age, the LAR decreases considerably with increasing age at start of screening, being 0.06%, 0.04%, and 0.025% when starting at 40, 45, or 50 years, respectively. The corresponding benefit-risk ratios are about 25, 35, and 45, respectively. Changing the upper screening age to 75 has little impact on the benefit-risk ratio.

**Conclusion:**

Extending the age limits in MSP to women starting from 45 years and up to 75 years is justified from the radiation perspective since the benefit substantially outweighs the radiation risk. Based on our report, the MSP has also been approved for women age 70 to 75 in Germany as of February 2024, while it is still pending for younger women.

**Key Points:**

- Screening can reduce breast cancer mortality in women age 45–49 and 70–75.
- As a result, more women can benefit from mammography screening programs.
- The downside for older women is more overdiagnoses.
- Younger women face a higher radiation risk.

**Citation Format:**

- Hunger T, Nekolla EA, Wanka-Pail E et al. Extending the Age Range in Mammography Screening: A Benefit-Risk Assessment from a Radiation Protection Perspective. Rofo 2026; 198: 164–172

## Zusammenfassung

**Hintergrund:**

Mammografie-Screening-Programme (MSP) für Frauen im Alter von 50 bis 69 Jahren sind in Deutschland und Europa etabliert. Einige der Studien, welche die Evidenzbasis für diese Programme bilden, schlossen auch Frauen ein, die jünger oder älter als diese Zielpopulation waren. Ziel unserer Studie war es, zu bewerten, ob das Screening auch für Frauen, die nicht der ursprünglichen Altersgruppe des MSP entsprechen, mehr Nutzen als Schaden bringt.

**Methoden:**

Eine systematische Übersichtsarbeit mit Metaanalyse der randomisierten kontrollierten Studien (RCTs) wurde durchgeführt, um die Gesamt- und Brustkrebssterblichkeit bei Frauen ab 70 Jahren und unter 50 Jahren zu untersuchen. Das strahlenassoziierte Lebenszeitrisiko (LAR) wurde anhand eines modifizierten Risikomodells des BEIR-Komitees unter Verwendung aktueller Krebs- und Sterbetafeldaten für eine weibliche deutsche Bevölkerung abgeschätzt.

**Ergebnisse:**

Zwei RCTs mit 33268 Frauen im Alter von mindestens 70 Jahre und acht RCTs mit 394080 Frauen im Alter von 39–49 Jahren wurden eingeschlossen. Die relative Reduktion der Brustkrebssterblichkeit betrug 28% (Risk Ratio (RR) = 0,72; 95%-Konfidenzintervall (KI): 0,54–0,95) bzw. 18% (RR = 0,82; 95%-KI: 0,71–0,96). Der Anteil der Überdiagnosen wird bei älteren Frauen auf 19%, also höher als bei jüngeren Frauen, geschätzt. Bei zweijährlichem Screening bis 69 ab einem Alter von unter 50 Jahren nimmt das LAR erheblich mit zunehmendem Alter bei Screening-Beginn ab und beträgt ca. 0,06%, 0,04% bzw. 0,025%, wenn ab 40, 45 bzw. 50 Jahren gescreent wird. Die zugehörigen Werte für das Nutzen-Risiko-Verhältnis liegen bei etwa 25, 35 bzw. 45. Eine Erhöhung des oberen Screening-Alters auf 75 Jahre hat wenig Einfluss auf das Nutzen-Risiko-Verhältnis.

**Schlussfolgerung:**

Die Erweiterung der Altersgrenzen in einem MSP auf Frauen ab 45 Jahren und bis 75 Jahren ist aus Strahlenschutzperspektive gerechtfertigt, da der Nutzen das Strahlenrisiko deutlich überwiegt. Basierend auf unserem Bericht wurde das MSP in Deutschland im Februar 2024 rechtlich auch für Frauen von 70–75 Jahren zugelassen, wohingegen die Zulassung für jüngere Frauen noch aussteht.

**Kernaussagen:**

- Früherkennung kann die Brustkrebssterblichkeit bei Frauen zwischen 45–49 und 70–75 Jahren reduzieren.
- Mehr Frauen können dadurch von Mammografie-Screening-Programmen profitieren.
- Nachteilig für ältere Frauen sind mehr Überdiagnosen.
- Jüngere Frauen haben ein höheres Strahlenrisiko.

## Introduction

Breast cancer is the most frequent cancer in women in Germany, affecting approximately one in eight women during their lifetime 1 . The median age at diagnosis is 65 years, with 40% of cases diagnosed in women after their 70 ^th^ birthday 1 . Age-standardized incidence rates increased in most European countries, including Germany, until the early 2000s. Since then, this trend has slowed down or stagnated. Age-standardized breast cancer death rates in the European Union (EU) declined with a percent change of –4.3% in Germany between 2012 and 2017 2 to about 22 per 100,000 women in 2022 3 . This success can be attributed to improved treatment, but also to early detection 4 .

National mammography screening programs (MSP) have been introduced in Europe starting as early as 1989 with slight differences between countries. Sweden, for example, offers biennial screening from 40 to 74 years and the Netherlands from 50 to 75 years. The European Commission (EC) first published European guidelines for quality assurance in breast cancer screening and diagnosis in 2006 5 . In 2009, a quality-assured MSP for women age 50 to 69 was fully implemented in Germany. Nevertheless, there is evidence for opportunistic screening outside the organized program among both eligible women and women beyond the recommended age of the MSP 6 .

The introduction of MSP in Europe was based on randomized controlled trials (RCT) providing primarily evidence for the benefit of breast cancer screening for postmenopausal women within the age range of 50–70 years. However, some of these trials also included women from younger and older age groups. The present study therefore aimed to assess the benefits and harms of mammography screening – with particular attention to X-ray–associated risks – in women outside the originally defined age range of the German MSP (50–69 years), in order to provide a scientific basis for extending eligibility. Screening of women age 70–75 has already been implemented based on our earlier scientific report 7 , while a decision regarding women age 45–49 is still pending.

## Materials & Methods

### Systematic literature review of benefits and harms

We conducted a systematic literature review in compliance with established standards 8 . The electronic databases Medline and Cochrane CENTRAL were searched using thesaurus and free-text terms for breast cancer, population screening, and X-ray mammography to identify studies on mammography screening for breast cancer. The last date of the database query was March 22, 2024. Additionally, a hand search of bibliographies of eligible publications was performed. Moreover, study registers were searched for ongoing trials.

Identified articles were included in the evidence synthesis if they related to RCTs or systematic reviews of RCTs that reported results on breast cancer specific and all-cause mortality. Secondary outcomes of interest were breast cancer incidence and stage distribution; diagnostic follow-up procedures in false-positives, both invasive and non-invasive; complications in follow-up procedures; overdiagnosis; and quality of life. Studies were independently selected by two experienced authors in a two-step approach. This involved an initial review of title and abstract of all database hits followed by thorough assessment of the relevant full-text publications. The methodological quality of the studies and their potential for bias were assessed with the Cochrane risk of bias tool 9 .

To compare outcomes between the screening and control groups across the included studies, meta-analyses were performed for breast cancer specific and all-cause mortality separately for women under 50 years and over 70 years using the Cochrane Review Manager software version 5.4 10 . Results for secondary outcomes derived from the included studies were summarized narratively.

### Estimation of radiation risk and benefit

The assessment of the radiation risk associated with an MSP is based on a modified risk approach of the Biological Effects of Ionising Radiation (BEIR) VII Committee 11 (see 12 for details). Two different models are used: a relative risk model based on data from the Life Span Study (LSS) of atomic bomb survivors (“LSS model”) and an absolute risk model from a meta-analysis by Preston et al. 13 (“meta-analysis model”), in which data of studies of medically exposed women were also considered. In both models a linear dose-response relationship and an age dependency are assumed (decreasing radiation risk with increasing age). The meta-analysis model predicts a lower radiation risk than the LSS model and is favored by the BEIR VII committee, as data not only from the Japanese LSS but also from Western cohorts were included in the risk modelling.

The radiation-related excess risk of developing or dying from cancer by the end of life following radiation exposure is referred to as lifetime attributable risk (LAR). The BEIR VII report provides outdated LAR estimates for the US population based on US cancer rates and mortality tables from the 1990s. In the present paper, current age-specific LARs were estimated for a German female population. A minimum latency period of five years until the clinical manifestation of a radiation-associated cancer and a dose and dose rate effectiveness factor, DDREF, of 1 was assumed for exposures resulting from X-ray mammography. The DDREF is “a judged factor that generalizes the usually lower biological effectiveness (per unit of dose) of radiation exposures at low doses and low dose rates as compared with exposures at high doses and high dose rates” 14 . The International Commission of Radiation Protection (ICRP) assumes a DDREF of 2. Competing (life-shortening) risks were accounted for in the estimation using current German life table data from the Federal Statistical Office 15 as well as baseline rates for breast cancer incidence and mortality 3 . In our approach, LARs were calculated using both the meta-analysis model and the LSS model. Deviating from the BEIR VII approach, both results were then incorporated in the overall estimate of the LAR with weighting factors of 0.7 and 0.3, respectively (for details see 12 ). This risk model approach and the assumption of a DDREF of 1 lead to a conservative estimate of the radiation risk. In the following, 2.7 mSv is assumed to be a representative mean organ equivalent dose to the breast for a bilateral screening mammography in two views 16 .

In order to estimate benefit risk ratios, two relevant aspects were taken into account when estimating the lifetime benefit of screening: (1) The intended prevention of breast cancer deaths only becomes effective with a time delay after a cancer is detected through screening, and the benefit persists for a certain period after screening has been ended. (2) In accordance with the estimate of hypothetical radiation-related deaths, competing causes of death were considered with regard to breast cancer deaths prevented by screening.

## Results

### Benefits and harms

**Fig. 1:**
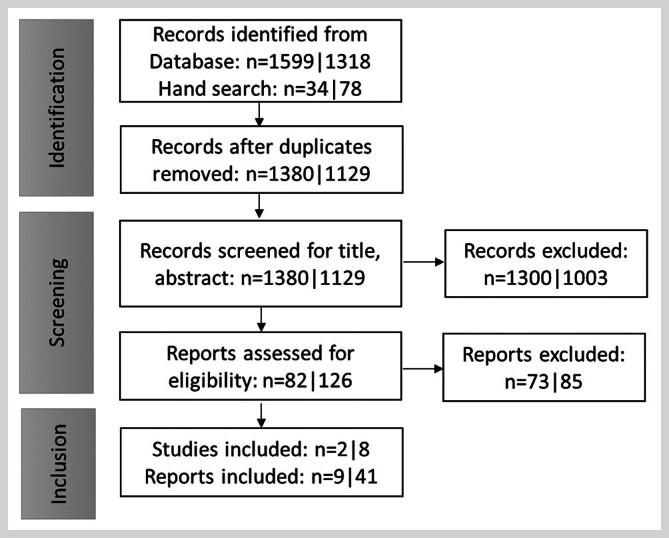
Flow diagram of literature selection for women ≥ 70 | < 50 years.

The literature search yielded over 2,500 records, and after applying the inclusion and exclusion criteria, we included eight individual RCTs. Two of them (Malmö I 17 and Two-County 18 ) report results for women 70 years and older in nine articles, and all eight RCTs (Canadian National Breast Screening Study (CNBSS) 19 , Edinburgh 20 , Göteborg 21 , Health Insurance Plan (HIP) 22 , Malmö I and II 23 , Stockholm 24 , Two-County 25 , and UK Age 26 ) report the results for women under 50 years in 41 articles (see supplement for a full list of included publications) ( Fig. 1 ). The main characteristics of the evaluated RCTs are summarized in Table 1 . They were performed in Europe and North America in the 1960s to 1990s and included women from 39 to 74 years. Overall, they provide data on 33,268 women who were at least 70 years at the start of the study or turned 70 during the active study period and on 394,080 women age 39–49 years at baseline. Screening was offered annually or biennially for two to nine rounds. Follow-up lasted at least six years and up to 30 years.

**Table:** **Table 1** Main characteristics of the RCTs included in the systematic review.

Study	Recruitment period	Study centres	Inclusion criteria	Randomization mode	Screening Interval [months]	Screening rounds	Follow-up [years]
CNBSS	1980–1985	15	40–49 years, no mammography in past 12 months, no history of breast cancer	individual	12	4–5 ^b^	25
Edinburgh	1978–1985	87	45–64 years, no history of breast cancer	cluster (practitioner)	24	2–4 ^c^	10–14 ^c^
Göteborg	1982–1984, 1987–1991 (controls)	1	39–59 years	cluster (date of birth)	18	5	24
HIP	1963–1966	n.i.	40–64 years ^a^	n.i.	12	4	18
Malmö I	1976–1978, 1992–1993 (controls)	n.i.	Birth year 1908–1932 ^a^	individual	18–24	6–9 ^d^	30
Malmö II	1978–1990, 1991–1994 (controls)	n.i.	Birth year 1933–1945 ^a^	individual	18–24	1–7 ^d^	23
Stockholm	1981–1985, 1985–1986 (controls)	n.i.	40–64 years	quasi-randomization by date of birth	28	2	26
Two-County	1977–1981	n.i.	40–74 years, no history of breast cancer	cluster (region)	24–33	2–5 ^b, d^	13
UK Age	1990–1996	23	39–41 years, no history of breast cancer	individual	12	8	23
^a^ women with history of breast cancer at baseline were excluded from analysis, ^b^ dependent on recruitment date, ^c^ cohort dependent, ^d^ age dependent; n.i.: no information							

Only one of the eight trials (UK Age) was considered to have a low risk of bias, the others either lacked an adequate randomization process or did not provide sufficient details about it. Three studies (Stockholm, Two-County, CNBSS) were judged to be at high risk of bias.

**Fig. 2:**
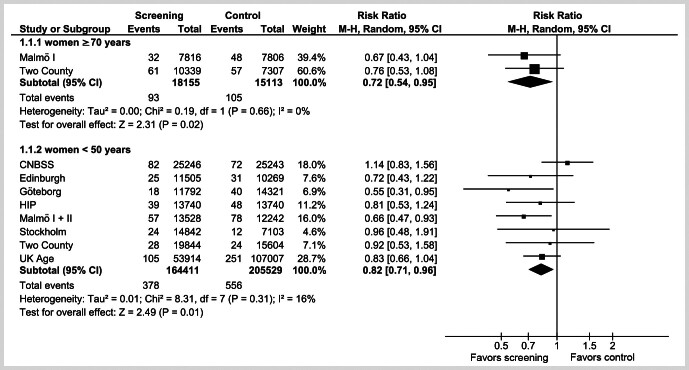
Forest Plot of breast cancer mortality in women ≥ 70 years (upper panel) and women < 50 years (lower panel) after about 10 years of follow-up. Squares with bars show the point estimates of individual studies with their confidence intervals. The size of the squares represents the weighting of the study. The diamond symbolizes the pooled effect of several studies.

During a 10-year follow-up period, 93 women (51 per 10,000) age 70 years and older died from breast cancer in the screening group, compared to 105 (70 per 10,000) in the control group. The meta-analysis resulted in a statistically significant relative reduction in breast cancer mortality of 28% (risk ratio (RR) = 0.72; 95% confidence interval (CI): 0.54–0.95) ( Fig. 2 ). In women under 50 years, 378 (23 per 10,000) died of breast cancer in the screening group and 556 (27 per 10,000) in the control group. This resulted in a statistically significant relative reduction in breast cancer mortality of 18% (RR = 0.82; 95%-CI: 0.71–0.96) ( Fig. 2 ). Additional sensitivity analyses showed that the reduction in breast cancer mortality was larger in studies with low or moderate risk of bias (RR = 0.75; 95%-CI: 0.64–0.88) and failed to reach statistical significance in studies with high risk of bias (RR = 1.06; 95%-CI: 0.82–1.37) (for details see scientific report 27 ). Analyses of age subgroups indicated a consistent effect for women age 39–44 years and 45–49 years, respectively (see 27 ). There was no effect of mammography screening on all-cause mortality both in women 70 years and older (RR = 0.99; 95%-CI: 0.91–1.07) and in women under 50 years (RR=1.01; 95%-CI: 0.98–1.05) (see 7 27 ).

Information on the tumor stage at diagnosis was limited for both age groups. One study with 143 breast cancer cases in women over 70 years reported a higher percentage of tumors diagnosed at stage I in the screening group compared to the control group (62.2% versus 37.3%, p<0.05) and fewer tumors diagnosed at stages III and IV (3.3% versus 9.8%, not statistically significant) 28 . For women under 50 years, data from six studies showed higher rates of carcinoma in-situ diagnoses in the screening group, but no statistically significant difference in the distribution of stages I to IV between the study groups (details see 27 ).

The frequency of interval cancers varies with different lengths of intervals between the mammograms in the studies. One study providing mammography every 24 months for women under 50 years reported 35.5% of cancers diagnosed between screening rounds 29 . In women over 70 years this percentage was only 6% in a study with a screening interval of 18–24 months 30 .

An estimate of overdiagnosis, based on the excess incidence in the screening group, suggests that up to 19% of breast cancers diagnosed in women of 70 years and older in the screening group are overdiagnoses that pose no threat and would not become clinically significant during their lifetime 7 . In women under 50 years the estimates of overdiagnosis vary between studies with a median of 14%. The available data on other negative effects of screening, such as false-positive results, unnecessary biopsies, and impacts on quality of life, are insufficient to provide definitive conclusions.

### Radiation risk and benefit-risk assessment

**Fig. 3:**
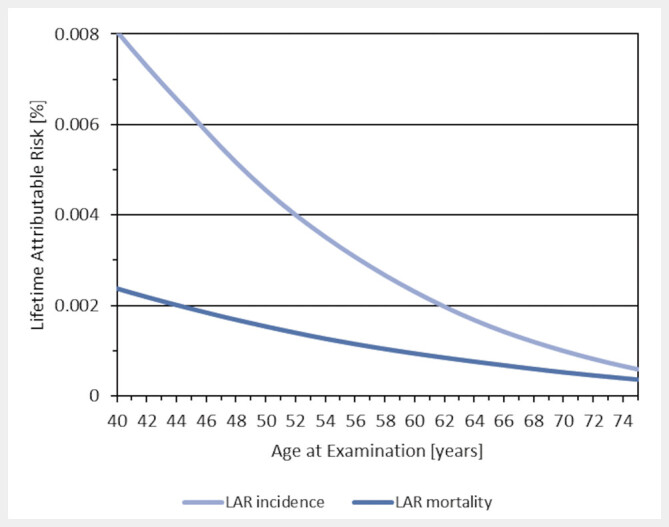
Lifetime attributable risk (LAR) per screening round according to our risk approach as a function of age at examination assuming a mean glandular dose of 2.7 mSv for breast cancer (lower line: mortality; upper line: incidence). Since the risk depends linearly on dose, estimates can be adjusted for other doses.

**Fig. 4:**
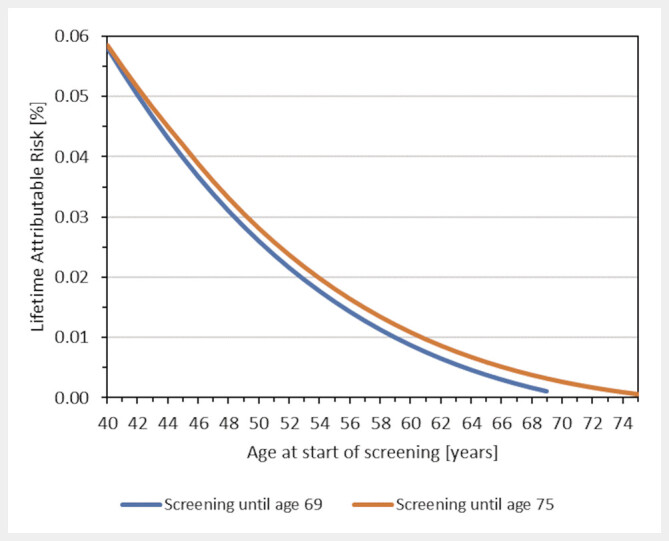
Lifetime attributable risk (LAR) for breast cancer incidence as a function of age at start of screening assuming biennial MSP until age 69 (lower line) or 75 (upper line) and a mean glandular dose of 2.7 mSv per round. Since the risk depends linearly on dose, estimates can be adjusted for other doses.

According to our approach, the LAR from a single mammogram decreases significantly with increasing age ( Fig. 3 ). If multiple participations in the MSP are considered, the age-specific LAR values add accordingly. For example, the LAR for breast cancer incidence in women who start at 50 and regularly participate in screening until 69 years is around 0.025%. If participation in the MSP continues for an additional three rounds beyond the age of 70, the LAR increases only slightly ( Fig. 4 ). Assuming biennial participation from an age below 50 years, the LAR decreases relatively steeply with increasing age at the start of screening and is about 0.06% and 0.04% when screening starts at the age of 40 or 45, respectively ( Fig. 4 ).

**Fig. 5:**
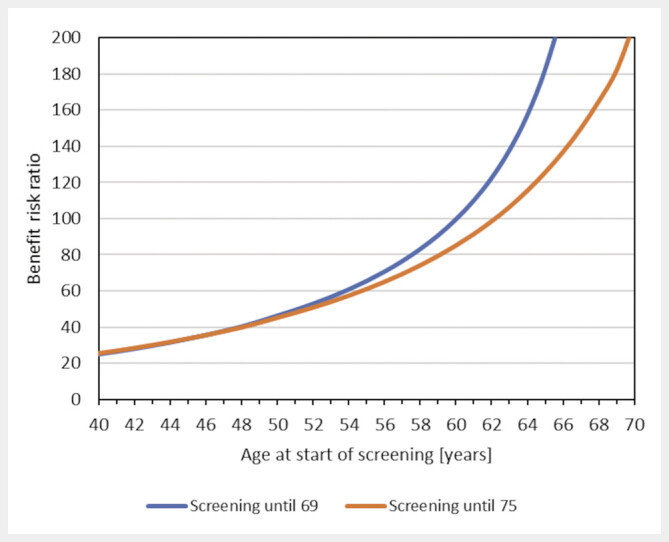
Benefit-risk ratio as a function of age at start of screening, assuming breast cancer mortality reductions of 18% (40–49 years), 25% (50–69 years), and 28% (70–75 years) with biennial screening (mean glandular dose = 2.7 mSv per round). Since the benefit-risk ratios are inversely proportional to dose, estimates can be adjusted for other radiation doses.

The benefit-risk ratio increases sharply with increasing age of entry, whereby the curve is almost linear at the beginning and becomes significantly steeper from the age of around 50 ( Fig. 5 ). For a screening start at the age of 40, 45, or 50 and regular screening until 75, the benefit-risk ratio is around 25, 35, or 45, respectively. The German Federal Office for Radiation Protection (BfS) usually requires a benefit-risk ratio of at least 10. For a screening start at the age of 60 (up to 69 years) or 62 (up to 75 years), the benefit-risk ratios exceed 100. The rapid upward trend in the benefit-risk ratio with age at the start of screening is due to the fact that the benefit decreases to a lesser extent than the excess radiation risk.

## Discussion

While early detection of breast cancer by mammography screening for women age 50 to 69 or 70 years is widely established, inclusion of younger and older women is not uniformly implemented across Europe. Our systematic literature review showed that both age groups also benefit from screening in terms of reducing breast cancer mortality. The relative benefit for women age 70–74 and under 50 years is 28% and 18%, respectively, and is thus comparable to that of 25% in women age 50–69 31 . The evidence from RCTs is limited for women over 70 years and relies only on 93 versus 105 breast cancer deaths in the mammography versus control groups (resulting in 18 fewer breast cancer deaths per 10,000 women). For women screened under 50 year, the relative benefit is only slightly lower as compared to the other age groups; however, yields lower absolute numbers of avoided breast cancer deaths (four fewer cases per 10,000), since breast cancer incidence is lower in younger women. It is of note that mortality reduction results in more life-years gained in this group.

The harms of screening also weigh differently for different age groups. Overdiagnosis is inherent in all cancer screening programs and difficult to quantify. In older women the potential for overdiagnosis must be considered carefully as limited remaining life expectancy and competing health risks increase the probability that small and early-stage cancers would not manifest clinically within their lifetimes. On the other hand, radiation risk from screening examinations in women over 70 is minimal, resulting in a favorable benefit-risk ratio.

In contrast, younger women are more susceptible to radiation effects and have a longer remaining lifespan during which potentially radiation related cancers might occur. The radiation risk associated with biennial mammography screening between the ages of 40–75 and 45–75 is higher by approximately a factor of 2 or 1.5, respectively, compared to screening between 50 and 75. Similar results are obtained when assuming a screening scenario with an upper age limit of 69 years, as the radiation risk for women over 70 is minimal. By contrast, the benefit-risk ratio is considerably lower for women who start screening at younger ages, not only because of the higher radiation exposure with more screening rounds, but especially because the radiation risk increases with decreasing age at exposure.

Our review includes RCTs that have already been used by the International Agency for Research on Cancer (IARC) for their initial recommendation for breast cancer screening in 2002 31 . The studies included were conducted in the 1960s to 1990s, and thus they do not reflect the current imaging technology, therapy standards (e.g. endocrine therapy, antibody therapy) or societal changes (e.g. reproductive behavior, higher life expectancy). Today, digital full-field mammography replaced screen-film mammography, and two-view imaging as well as double-reading have become standard practices in MSP. These advances have led to higher sensitivity, lower recall rates, and reduced radiation exposure 32 33 34 . Moreover, all studies but one had randomization issues or lacked sufficient detail for assessment. Randomization by clusters, e.g. geographical region, as well as pre-randomization examinations may have introduced bias in the study group allocation. Contamination by screening outside the study and offering screening to the control group after the active study period likely diluted the observed effects. The studies with high risk of bias slightly reduced the overall effect, leading to more conservative results.

Our review's results on breast cancer mortality reduction are consistent with those of other reviews, such as Canelo-Aybar et al. 35 , which is the evidence base for the breast cancer screening guidelines of the European Commission Initiative on Breast Cancer (ECIBC) 36 . The ongoing AgeX trial, which offers women age 47–49 years a single mammogram before starting regular screening from age 50–70, and an additional screen to women age 71–73 years, is expected to publish results in 2026 ( www.agex.uk ). This study will add to the findings from the present analysis, but it is unlikely to change the basic conclusion that mammography screening can reduce breast cancer mortality in women over 70 and under 50 years of age.

While most reviews focus on the benefits of mammography screening, we estimated the associated radiation risks and the benefit-risk ratios using updated age-specific risk models for various screening scenarios. A constant average organ equivalent dose to the breast of 2.7 mSv per mammographic examination was assumed. This value reflects a population-based median value derived from MSP data and includes women with varying breast sizes and densities. Although the breast dose tends to decrease with increasing age, the assumption of a constant dose value over the screening age range provides a reasonable approximation for risk modelling across the heterogeneous screening population. This approach also avoids overinterpretation of dose differences that are subject to considerable anatomical and technical variability. The results can be readily adapted to any other value of the glandular dose or to future findings regarding the benefits of breast cancer screening. It is important to note that the potential benefit for patients with screen-detected breast cancer would be immediate, whereas a radiation-induced cancer remains a hypothetical risk that would only manifest after a latency period of several years or even decades. The latency period is particularly relevant for participants who engage in screening at an older age.

Based on our study, the responsible German Federal Ministry declared that the German MSP is also permitted for women age 70 to 75 in February 2024 37 , and they are able to participate in the MSP since July 2024. It is to be expected that approval will also be granted for the 45 to 49 age group. Extending the age limits for mammography screening can only provide benefit on a population level, if a sufficient number of women follow the invitation. In the established MSP in Germany, just over 50% of eligible women participate 38 , which is considerably lower than the 70% recommended by the European guidelines 5 . Most women who were asked about their reasons for not following the screening invitation reported that they had received mammograms outside the national MSP 6 . This opportunistic screening does not meet the rigorous quality standards of the organized MSP, such as double reading of mammograms by experienced radiologists. Efforts have been made to increase participation 39 , and the recent legal authorization to screen older women might raise additional public awareness on the MSP.

Mammography is still the gold standard in breast imaging, despite its limitations in terms of sensitivity and false-positive rates in younger women and in those with very dense breast tissue 40 41 . Those women may not equally benefit from mammography screening and might be better screened by alternative or supplemental technologies, e.g. with digital breast tomosynthesis (DBT) 42 or magnetic resonance mammography 43 . In recent years, the use of DBT has become widespread in the diagnostic work-up of abnormal mammography findings and it may be suitable for early detection when combined with synthesized two-dimensional mammography. Studies indicate increased detection rates, especially of early stage and small cancers 44 45 46 47 48 , but also partly higher recalls for follow-up procedures 47 48 . The ECIBC 36 and the United States Preventive Services Task Force 49 recognize a potential role for DBT in breast cancer screening. In Germany DBT for breast cancer screening is currently under investigation by the BfS.

While our study highlights the benefits of more comprehensive screening, there is also evidence that de-escalation and personalization of screening may avoid overdiagnosis and overtreatment 50 51 52 . The ECIBC, for example, suggest an extended screening interval of three years for women age 70 years and older 36 . More research in this field is required, and the organizational challenges of restructuring population-wide established programs need to be reflected.

## Conclusion

Our study demonstrated that the relative benefit for women who are screened before age 50 and after age 69 is consistent with those of women age 50 to 69 years at screening. The associated radiation risk is low, resulting in a clearly positive benefit-risk ratio. Accordingly, the BfS recommends extending the age limits of the German MSP to 45 to 75 years to enable more women to benefit from screening. However, older women are at increased risk of overdiagnosis, and younger women are subject to a higher radiation-related risk. Therefore, women need to be adequately educated about both the potential benefits and the risks in order to make an informed decision.

## Supplementary Material

Supplementary Material Zusatzmaterial
