# Supplementary material for: Extending the Age Range in Mammography Screening: A Benefit-Risk Assessment from a Radiation Protection Perspective
Source: Rofo. 2025 Aug 28;198(2):164–72. doi: 10.1055/a-2674-5744 (PMC12851822; doi:10.1055/a-2674-5744)
Supplement: Supplementary file 1 — Supplementary Material [file 10-1055-a-2674-5744_26745909.pdf]

Supplement

S1. List of included publications in the systematic review

1. Alexander FE, Anderson TJ, Brown HK et al. 14 years of follow-up from the Edinburgh randomised trial of breast-cancer screening. Lancet 1999; 353(9168): 1903-1908. https://dx.doi.org/10.1016/s0140-6736(98)07413-3
2. Alexander FE, Anderson TJ, Brown HK et al. The Edinburgh Randomized Trial of Breast-Cancer Screening - Results after 10 Years of Follow-Up. Br J Cancer, 1994; 70(3): 542-548.
3. Andersson I and Janzon L. Reduced breast cancer mortality in women under age 50: updated results from the Malmö Mammographic Screening Program. J Natl Cancer Inst Monogr 1997; (22): 63-67. https://dx.doi.org/10.1093/jncimono/1997.22.63
4. Andersson I, Aspegren K, Janzon L et al. Mammographic screening and mortality from breast cancer: the Malmö mammographic screening trial. BMJ 1988; 297(6654): 943-948. https://dx.doi.org/10.1136/bmj.297.6654.943
5. Andersson I. Radiographic screening for breast carcinoma. I. Program and primary findings in 45--69 year old women. Acta Radiol Diagn (Stockh) 1981; 22(2): 185-194. https://dx.doi.org/10.1177/028418518102200213
6. Aron JL and Prorok PC. An analysis of the mortality effect in a breast cancer screening study. Int J Epidemiol 1986; 15(1): 36-43. https://dx.doi.org/10.1093/ije/15.1.36
7. Baines CJ, To T, Miller AB. Revised estimates of overdiagnosis from the Canadian National Breast Screening Study. Prev Med 2016; 90: 66-71. https://dx.doi.org/10.1016/j.ypmed.2016.06.033
8. Bjurstam N, Björneld L, Duffy SW et al. The Gothenburg breast screening trial: first results on mortality, incidence, and mode of detection for women ages 39-49 years at randomization. Cancer 1997; 80(11): 2091-2099.
9. Bjurstam N, Björneld L, Warwick J et al. The Gothenburg Breast Screening Trial. Cancer 2003; 97(10): 2387-2396. https://dx.doi.org/10.1002/cncr.11361
10. Bjurstam NG, Bjorneld LM, Duffy SW. Updated results of the Gothenburg Trial of Mammographic Screening. Cancer 2016; 122(12): 1832-1835. https://dx.doi.org/10.1002/cncr.29975
11. Chu KC, Smart CR and Tarone RE. Analysis of breast cancer mortality and stage distribution by age for the Health Insurance Plan clinical trial. J Natl Cancer Inst 1988; 80(14): 1125-1132. https://dx.doi.org/10.1093/jnci/80.14.1125
12. Duffy S, Vulkan D, Cuckle H et al. Annual mammographic screening to reduce breast cancer mortality in women from age 40 years: long-term follow-up of the UK Age RCT. Health Technol Assess 2020; 24(55): 1-24. https://dx.doi.org/10.3310/hta24550
13. Duffy SW, Tabar L, Vitak B et al. The Swedish Two-County Trial of mammographic screening: cluster randomisation and end point evaluation. Ann Oncol 2003; 14(8): 1196-1198. https://dx.doi.org/10.1093/annonc/mdg322
14. Duffy SW, Vulkan D, Cuckle H et al. Effect of mammographic screening from age 40 years on breast cancer mortality (UK Age trial): final results of a randomised, controlled trial. Lancet Oncol 2020; 21(9): 1165-1172. https://dx.doi.org/10.1016/s1470-2045(20)30398-3
15. Fagerberg G, Baldetorp L, Grontoft O et al. Effects of repeated mammographic screening on breast cancer stage distribution. Results from a randomised study of 92 934 women in a Swedish county. Acta Radiol Oncol 1985; 24(6): 465-73.
16. Frisell J and Lidbrink E. The Stockholm Mammographic Screening Trial: Risks and benefits in age group 40-49 years. J Natl Cancer Inst Monogr 1997; (22): 49-51. https://dx.doi.org/10.1093/jncimono/1997.22.49

17. Frisell J, Eklund G, Hellstrom L et al. Randomized study of mammography screening--preliminary report on mortality in the Stockholm trial. Breast Cancer Res Treat 1991; 18(1): 49-56. https://dx.doi.org/10.1007/BF01975443
18. Frisell J, Eklund G, Hellstrom Land Somell A. Analysis of interval breast carcinomas in a randomized screening trial in Stockholm. Breast Cancer Res Treat 1987; 9 (3): 219-25.
19. Frisell J, Glas U, Hellström L et al. Randomized mammographic screening for breast cancer in Stockholm. Design, first round results and comparisons. Breast Cancer Res Treat 1986; 8(1): 45-54. https://dx.doi.org/10.1007/bf01805924
20. Frisell J, Lidbrink E, Hellström L et al. Followup after 11 years--update of mortality results in the Stockholm mammographic screening trial. Breast Cancer Res Treat 1997; 45(3): 263-270. https://dx.doi.org/10.1023/a:1005872617944
21. Habbema JD, van Oortmarssen GJ, van Putten DJ et al. Age-specific reduction in breast cancer mortality by screening: an analysis of the results of the Health Insurance Plan of Greater New York study. J Natl Cancer Inst 1986; 77(2): 317-320.
22. Johns LE, Moss SM, Age Trial Management Group. False-positive results in the randomized controlled trial of mammographic screening from age 40 ("Age" trial). Cancer Epidemiol Biomarkers Prev 2010; 19(11): 2758-2764. https://dx.doi.org/10.1158/1055-9965.EPI-10-0623
23. Lidbrink E, Elfving J, Frisell J and Jonsson E. Neglected aspects of false positive findings of mammography in breast cancer screening: analysis of false positive cases from the Stockholm trial. BMJ 1996; 312(7026): 273-6.
24. Miller AB, Baines CJ, To T et al. Canadian National Breast Screening Study: 1. Breast cancer detection and death rates among women aged 40 to 49 years. CMAJ 1992; 147(10): 1459-1476.
25. Miller AB, To T, Baines CJ and Wall C. The Canadian National Breast Screening Study: update on breast cancer mortality. J Natl Cancer Inst Monogr 1997; 22: 37-41.
26. Miller AB, To T, Baines CJ et al. The Canadian National Breast Screening Study-1: breast cancer mortality after 11 to 16 years of follow-up. A randomized screening trial of mammography in women age 40 to 49 years. Ann Intern Med 2002; 137(5 Part 1): 305-312. https://dx.doi.org/10.7326/0003-4819-137-5_part_1-200209030-00005
27. Moss S, Thomas I, Evans A et al. Randomised controlled trial of mammographic screening in women from age 40: results of screening in the first 10 years. Br J Cancer 2005; 92(5): 949-954. https://dx.doi.org/10.1038/sj.bjc.6602396
28. Moss SM, Cuckle H, Evans A et al. Effect of mammographic screening from age 40 years on breast cancer mortality at 10 years follow-up: a randomised controlled trial. Lancet 2006; 368(9552): 2053-2060. https://dx.doi.org/10.1016/s0140-6736(06)69834-6
29. Moss SM, Wale C, Smith R et al. Effect of mammographic screening from age 40 years on breast cancer mortality in the UK Age trial at 17 years follow-up: a randomised controlled trial. Lancet Oncol 2015; 16(9): 1123-1132. https://dx.doi.org/10.1016/s1470-2045(15)00128-x
30. Narod SA, Sun P, Wall C et al. Impact of screening mammography on mortality from breast cancer before age 60 in women 40 to 49 years of age. Curr Oncol 2014; 21(5): 217-221. https://dx.doi.org/10.3747/co.21.2067
31. Nyström L, Bjurstam N, Jonsson H et al. Reduced breast cancer mortality after 20+ years of follow-up in the Swedish randomized controlled mammography trials in Malmö, Stockholm, and Göteborg. Journal of medical screening 2017; 24(1): 34-42.
32. Roberts MM, Alexander FE, Anderson TJ et al. Edinburgh trial of screening for breast cancer: mortality at seven years. Lancet 1990; 335(8684): 241-246. https://dx.doi.org/10.1016/0140-6736(90)90066-e

33. Shapiro S, Venet W, Strax P et al. Ten- to fourteen-year effect of screening on breast cancer mortality. J Natl Cancer Inst 1982; 69(2): 349-355.
34. Shapiro S. Evidence on screening for breast cancer from a randomized trial. Cancer 1977; 39(6 Suppl): 2772-2782. https://dx.doi.org/10.1002/1097-0142(197706)39:6<2772::aidcncr2820390665>3.0.co;2-k
35. Shapiro S. Periodic screening for breast cancer: the HIP Randomized Controlled Trial. Health Insurance Plan. J Natl Cancer Inst Monogr 1997; (22): 27-30. https://dx.doi.org/10.1093/jncimono/1997.22.27
36. Tabar L, Duffy SW, Yen MF et al. All-cause mortality among breast cancer patients in a screening trial: support for breast cancer mortality as an end point. J Med Screen 2002; 9(4): 159-162. https://dx.doi.org/10.1136/jms.9.4.159
37. Tabar L, Fagerberg CJ, Gad A et al. Reduction in mortality from breast cancer after mass screening with mammography. Randomised trial from the Breast Cancer Screening Working Group of the Swedish National Board of Health and Welfare. Lancet 1985; 1(8433): 829-832. https://dx.doi.org/10.1016/s0140-6736(85)92204-4
38. Tabar L, Fagerberg G, Chen HH et al. Efficacy of breast cancer screening by age. New results from the Swedish Two-County Trial. Cancer 1995; 75(10): 2507-2517. https://dx.doi.org/10.1002/1097-0142(19950515)75:10<2507::aid-cncr2820751017>3.0.co;2-h
39. Tabar L, Fagerberg G, Chen HH et al. Screening for breast cancer in women aged under 50: mode of detection, incidence, fatality, and histology. J Med Screen 1995: 2(2): 94-8.
40. Tabar L, Fagerberg G, Duffy SW et al. The Swedish two county trial of mammographic screening for breast cancer: recent results and calculation of benefit. J Epidemiol Community Health 1989; 43(2): 107-114. https://dx.doi.org/10.1136/jech.43.2.107
41. Tabár L, Vitak B, Chen HH et al. The Swedish Two-County Trial twenty years later. Updated mortality results and new insights from long-term follow-up. Radiol Clin North Am 2000; 38(4): 625-651. https://dx.doi.org/10.1016/s0033-8389(05)70191-3
